# Signal stability of Cy3 and Cy5 on antibody microarrays

**DOI:** 10.1186/1477-5956-4-21

**Published:** 2006-10-11

**Authors:** Qiang Gu, Thamil Mani Sivanandam, Caroline Aehyun Kim

**Affiliations:** 1 Department of Neurobiology and Anatomy, Wake Forest University School of Medicine, Medical Center Boulevard, Winston-Salem, North Carolina 27157, USA

## Abstract

**Background:**

The antibody microarray technique is a newly emerging proteomics tool for differential protein expression analyses that uses fluorescent dyes Cy 3 and Cy 5. Environmental factors, such as light exposure, can affect the signal intensity of fluorescent dyes on microarray slides thus, it is logical to scan microarray slides immediately after the final wash and drying processes. However, no research data are available concerning time-dependent changes of fluorescent signals on antibody microarray slides to this date. In the present study, microarray slides were preserved at -20°C after regular microarray experiments and were rescanned at day 10, 20 and 30 to evaluate change in signal intensity.

**Results:**

Fluorescent intensities of microarray spots were detected using a confocal laser scanner after the experiment at day 0, and re-examined at day 10, 20 and 30, respectively. Fluorescent intensities of rescanned microarray spots did not show significant changes when compared with those scanned immediately after standard microarray experiments.

**Conclusion:**

Microarray slides can be preserved and rescanned multiple times using a confocal laser scanner over a period of days or weeks.

## Background

Antibody microarray analyses of protein expression levels represent a new trend of cutting-edge proteomics research [1-6], and have been increasingly utilized in studies of normal and pathological conditions [7-22]. The technique has several distinct advantages. First, compared to the cDNA microarray analysis, antibody microarrays detect differential gene expression at the protein level. Although cDNA microarray analyses have generated a large database concerning gene expression patterns, most of these studies have focused on gene expression at the mRNA level only, with the assumption that the relative mRNA levels represent the relative levels of proteins. Since there is often a poor correlation between mRNA levels and protein levels [23-28], this assumption may not be true for many of the genes. Second, the antibody microarray technique is more sensitive compared to gel electrophoreses. With microarray technology, protein levels can be detected in the low pg/ml range. This allows the measurement of the expression of both small and large molecular weight proteins simultaneously, regardless of their isoelectric points. In addition, some protein extraction buffers used for antibody microarray experiments contain non-denaturing detergents in order to keep the proteins in their native state. While gel electrophoreses usually separate denatured proteins for expression analyses, antibody microarrays can measure relative abundance of naïve undenatured proteins.

The vast majority of antibody microarray experiments conducted so far used the fluorescent dyes Cy3 and Cy5 for protein labelling. In general, fluorescent dyes are sensitive to light exposure as well as other environmental factors such as water, high temperature, alkali, and alcohol. Recent evidence indicates that even ozone levels in the laboratory atmosphere could affect fluorescent dyes on microarrays [29]. In order to avoid or to minimize the effects of these risk factors, it is appropriate to scan microarray slides instantly upon finishing the final wash and drying processes without unnecessary delays. However, no data are currently available concerning time-dependent changes of fluorescent signals on antibody microarray slides. Furthermore, the published research on repeated scans of antibody or protein microarrays is scant. Although repeated scans of cDNA microarrays have been conducted [30-35], it is not known whether amine-coupled fluorescent dyes on antibody/protein microarrays behave in the same way as those nucleotide-coupled fluorescent dyes on cDNA microarrays. In addition, previous studies conducted multiple scans of cDNA microarrays sequentially, usually within minutes. Therefore, it is also unknown whether prolonged intervals between scans in the range of days or weeks could modify the signal intensity of fluorescent dyes on antibody or protein microarrays. Furthermore, previous rescan studies were conducted using different laser power levels or photomultiplier tube (PMT) gains rather than using a consistent setting of the laser power and PMT. It is expected that different settings of the laser power and PMT generated different signal intensities. Whether the same setting of the laser power and PMT can generate consistent intensity outcome over time has not been demonstrated. Using a confocal laser scanner to minimize photo-bleaching effects, we scanned microarrays slides at day 0, 10, 20 and 30, respectively. After regular antibody microarray experiments, the microarray slides were stored in a laboratory freezer at -20°C. Fluorescent intensities of microarray spots at these time points were then quantified and compared. Our results indicate no significant changes in intensities of both Cy3 and Cy5 signals over the examined period.

## Results and discussion

Clontech™ antibody microarrays were used in this study, which had 1024 microarray spots on each slide. Among them, 6 spots were printed with fluorescence-labeled albumin and served as positive controls, whereas 4 spots were printed with non-labeled albumin and served as negative controls. For all microarray slides, the negative control spots had similar intensities as those of background signals. These 10 control spots were not included in the final microarray spot analyses. The remaining 1014 spots on each microarray slide were followed up throughout the entire length of the study. In order to keep the same experimental condition, the following steps were taken: when a storage vial containing the microarray slide was taken out of the freezer, it was kept at room temperature for 30 minutes; and to keep the scan condition consistent, the same strength of the laser power (100%) and PMT (65%) were used for all scans of the microarray slides. No spot on the microarray slides was saturated using these settings of the laser power and PMT, so that unchanged intensities of microarray spots due to saturation could be excluded in the present study.

For each microarray spot, the signal intensity was measured 4 times, at day 0 (I_0_), day 10 (I_10_), day 20 (I_20_), and day 30 (I_30_), respectively. Because I_0 _of each microarray spot had a different value, we set I_0 _as the reference, and calculated the ratio of I_10_/I_0_, I_20_/I_0_, and I_30_/I_0_, respectively, for each of the 1014 spots on the microarray slide. If the fluorescent signals on a microarray slide decrease over time, the ratio value of I_10_/I_0_, I_20_/I_0_, and I_30_/I_0 _should become smaller and smaller. Fourteen slides were examined for Cy3 and for Cy5, respectively.

**Figure 1:**
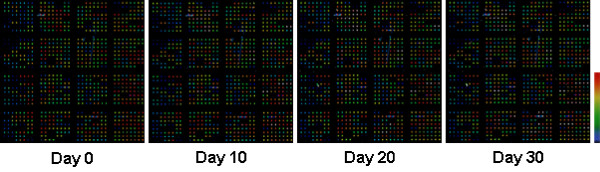
Examples of Cy3 signals on a microarray slide scanned atday 0, 10, 20, and 30, respectively. At day 0, the spot intensities on this slide range from 261 to 46409, with an average background intensity of 366 versus an average intensity of 488 for the 4 negative control spots. The scale bar on the right indicates color-coded signal intensities.

Figure 1 shows an example of Cy3 signals on a microarray slide at day 0, 10, 20, and 30, respectively. There was no apparent decrease of Cy3 signal intensities.

Quantitative analyses of all Cy3 spots (summarized in Table 1) further suggest that there was no statistically significant change in intensities of Cy3 signals over the examined time frame (p > 0.05, ANOVA).

**Table 1:** Summary of ratio comparisons of Cy3 spots.

**Slide Number**	I10/I0¯ MathType@MTEF@5@5@+=feaafiart1ev1aaatCvAUfKttLearuWrP9MDH5MBPbIqV92AaeXatLxBI9gBamXvP5wqSXMqHnxAJn0BKvguHDwzZbqegyvzYrwyUfgarqqtubsr4rNCHbGeaGqiA8vkIkVAFgIELiFeLkFeLk=iY=Hhbbf9v8qqaqFr0xc9pk0xbba9q8WqFfeaY=biLkVcLq=JHqVepeea0=as0db9vqpepesP0xe9Fve9Fve9GapdbaqaaeGacaGaaiaabeqaamqadiabaaGcbaWaa0aaaeaaimqacaWFjbWaaSbaaSqaaiaa=fdacaWFWaaabeaakiaa=9cacaWFjbWaaSbaaSqaaiaa=bdaaeqaaaaaaaa@41C2@	I20/I0¯ MathType@MTEF@5@5@+=feaafiart1ev1aaatCvAUfKttLearuWrP9MDH5MBPbIqV92AaeXatLxBI9gBamXvP5wqSXMqHnxAJn0BKvguHDwzZbqegyvzYrwyUfgarqqtubsr4rNCHbGeaGqiA8vkIkVAFgIELiFeLkFeLk=iY=Hhbbf9v8qqaqFr0xc9pk0xbba9q8WqFfeaY=biLkVcLq=JHqVepeea0=as0db9vqpepesP0xe9Fve9Fve9GapdbaqaaeGacaGaaiaabeqaamqadiabaaGcbaWaa0aaaeaaimqacaWFjbWaaSbaaSqaaiaa=jdacaWFWaaabeaakiaa=9cacaWFjbWaaSbaaSqaaiaa=bdaaeqaaaaaaaa@41C3@	I30/I0¯ MathType@MTEF@5@5@+=feaafiart1ev1aaatCvAUfKttLearuWrP9MDH5MBPbIqV92AaeXatLxBI9gBamXvP5wqSXMqHnxAJn0BKvguHDwzZbqegyvzYrwyUfgarqqtubsr4rNCHbGeaGqiA8vkIkVAFgIELiFeLkFeLk=iY=Hhbbf9v8qqaqFr0xc9pk0xbba9q8WqFfeaY=biLkVcLq=JHqVepeea0=as0db9vqpepesP0xe9Fve9Fve9GapdbaqaaeGacaGaaiaabeqaamqadiabaaGcbaWaa0aaaeaaimqacaWFjbWaaSbaaSqaaiaa=ndacaWFWaaabeaakiaa=9cacaWFjbWaaSbaaSqaaiaa=bdaaeqaaaaaaaa@41C4@
1	1.037 ± 0.004	1.086 ± 0.005	1.157 ± 0.006
2	1.008 ± 0.017	1.047 ± 0.004	1.087 ± 0.004
3	0.986 ± 0.003	1.025 ± 0.003	1.006 ± 0.002
4	1.021 ± 0.002	1.093 ± 0.002	0.988 ± 0.002
5	0.973 ± 0.007	1.006 ± 0.008	1.071 ± 0.009
6	1.044 ± 0.006	1.121 ± 0.007	1.104 ± 0.007
7	0.991 ± 0.008	0.990 ± 0.003	0.990 ± 0.003
8	0.982 ± 0.003	0.929 ± 0.003	0.948 ± 0.003
9	1.039 ± 0.004	1.051 ± 0.004	1.060 ± 0.004
10	1.055 ± 0.004	1.064 ± 0.006	1.001 ± 0.004
11	1.084 ± 0.009	1.142 ± 0.004	1.075 ± 0.004
12	1.066 ± 0.003	1.117 ± 0.012	1.026 ± 0.003
13	1.135 ± 0.007	1.138 ± 0.006	1.083 ± 0.021
14	0.985 ± 0.004	1.101 ± 0.003	1.114 ± 0.014
**Average**	**1.029 ± 0.013**	**1.065 ± 0.018**	**1.051 ± 0.017**

Each of the 1014 microarray spot was examined at day 0, 10, 20, and 30, respectively, and its intensities (I_0_, I_10_, I_20_, and I_30_) were measured. Then, the ratios of I_10_/I_0_, I_20_/I_0_, and I_30_/I_0 _for each microarray spot were generated. The average ratios of all 1014 spots on each individual slide at day 10 (I_10_/I_0_), day 20 (I_20_/I_0_), and day 30 (I_30_/I_0_) as well as the averaged value of all slides at day 10, 20 and 30 are shown (mean ± SEM).

**Figure 2:**
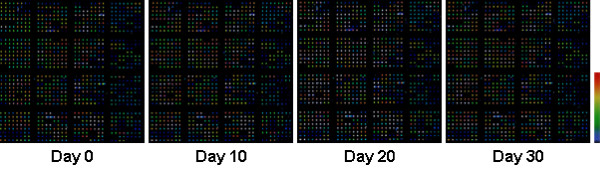
Examples of Cy5 signals on a microarray slide scanned at day 0, 10, 20, and 30, respectively. At day 0, the spot intensities on this slide range from 592 to 58229, with an average background intensity of 2504 versus an average intensity of 2512 for the 4 negative control spots. The scale bar on the right indicates color-coded signal intensities.

Figure 2 shows an example of Cy5 signals on a microarray slide at day 0, 10, 20, and 30, respectively. Like the case of Cy3, there was no apparent decrease of Cy5 signal intensities. Quantitative analyses of all Cy5 spots (summarized in Table 2) also suggest that there was no statistically significant change in intensities of Cy5 signals over the examined time frame (p > 0.05, ANOVA).

**Table 2:** Summary of ratio comparisons of Cy5 spots.

**Slide Number**	I10/I0¯ MathType@MTEF@5@5@+=feaafiart1ev1aaatCvAUfKttLearuWrP9MDH5MBPbIqV92AaeXatLxBI9gBamXvP5wqSXMqHnxAJn0BKvguHDwzZbqegyvzYrwyUfgarqqtubsr4rNCHbGeaGqiA8vkIkVAFgIELiFeLkFeLk=iY=Hhbbf9v8qqaqFr0xc9pk0xbba9q8WqFfeaY=biLkVcLq=JHqVepeea0=as0db9vqpepesP0xe9Fve9Fve9GapdbaqaaeGacaGaaiaabeqaamqadiabaaGcbaWaa0aaaeaaimqacaWFjbWaaSbaaSqaaiaa=fdacaWFWaaabeaakiaa=9cacaWFjbWaaSbaaSqaaiaa=bdaaeqaaaaaaaa@41C2@	I20/I0¯ MathType@MTEF@5@5@+=feaafiart1ev1aaatCvAUfKttLearuWrP9MDH5MBPbIqV92AaeXatLxBI9gBamXvP5wqSXMqHnxAJn0BKvguHDwzZbqegyvzYrwyUfgarqqtubsr4rNCHbGeaGqiA8vkIkVAFgIELiFeLkFeLk=iY=Hhbbf9v8qqaqFr0xc9pk0xbba9q8WqFfeaY=biLkVcLq=JHqVepeea0=as0db9vqpepesP0xe9Fve9Fve9GapdbaqaaeGacaGaaiaabeqaamqadiabaaGcbaWaa0aaaeaaimqacaWFjbWaaSbaaSqaaiaa=jdacaWFWaaabeaakiaa=9cacaWFjbWaaSbaaSqaaiaa=bdaaeqaaaaaaaa@41C3@	I30/I0¯ MathType@MTEF@5@5@+=feaafiart1ev1aaatCvAUfKttLearuWrP9MDH5MBPbIqV92AaeXatLxBI9gBamXvP5wqSXMqHnxAJn0BKvguHDwzZbqegyvzYrwyUfgarqqtubsr4rNCHbGeaGqiA8vkIkVAFgIELiFeLkFeLk=iY=Hhbbf9v8qqaqFr0xc9pk0xbba9q8WqFfeaY=biLkVcLq=JHqVepeea0=as0db9vqpepesP0xe9Fve9Fve9GapdbaqaaeGacaGaaiaabeqaamqadiabaaGcbaWaa0aaaeaaimqacaWFjbWaaSbaaSqaaiaa=ndacaWFWaaabeaakiaa=9cacaWFjbWaaSbaaSqaaiaa=bdaaeqaaaaaaaa@41C4@
1	1.115 ± 0.013	1.102 ± 0.013	1.054 ± 0.013
2	1.139 ± 0.005	1.162 ± 0.007	1.141 ± 0.006
3	1.103 ± 0.006	1.185 ± 0.006	0.999 ± 0.072
4	1.150 ± 0.022	1.153 ± 0.020	1.053 ± 0.012
5	1.054 ± 0.007	1.133 ± 0.008	1.180 ± 0.009
6	1.170 ± 0.007	1.220 ± 0.008	1.191 ± 0.008
7	1.120 ± 0.006	1.093 ± 0.006	1.070 ± 0.012
8	0.999 ± 0.011	0.970 ± 0.003	1.010 ± 0.082
9	1.030 ± 0.004	0.978 ± 0.004	0.943 ± 0.004
10	1.055 ± 0.006	1.005 ± 0.007	1.054 ± 0.009
11	0.959 ± 0.005	0.957 ± 0.037	0.868 ± 0.003
12	0.902 ± 0.002	0.844 ± 0.002	0.814 ± 0.003
13	1.080 ± 0.006	0.996 ± 0.006	1.025 ± 0.007
14	0.932 ± 0.002	0.797 ± 0.003	0.828 ± 0.003
**Average**	**1.058 ± 0.024**	**1.042 ± 0.037**	**1.016 ± 0.034**

Each of the 1014 microarray spot was examined at day 0, 10, 20, and 30, respectively, and its intensities (I_0_, I_10_, I_20_, and I_30_) were measured. Then, the ratios of I_10_/I_0_, I_20_/I_0_, and I_30_/I_0 _for each microarray spot were generated. The average ratios of all 1014 spots on each individual slide at day 10 (I_10_/I_0_), day 20 (I_20_/I_0_), and day 30 (I_30_/I_0_) as well as the averaged value of all slides at day 10, 20 and 30 are shown (mean ± SEM).

Microarray spot signal intensities can span a broad range from 0 to 65536 (= 2^16^). To examine whether microarray spots with different intensities behave differently over time, we divided the microarray spots into three groups according to their intensities: 1) lower range when I_0 _was lower than 20000, 2) middle range when I_0 _was between 20000 and 40000, and 3) higher range when I_0 _was higher than 40000, and calculated spot intensities in each group of the Cy3-labeled (N = 14 slides) and in each group of the Cy5-labeled (N = 14 slides) at day 0, 10, 20, and 30, respectively. Quantitative analyses did not show statistically significant changes in intensities over the examined time frame regardless of their intensities. (p > 0.05, ANOVA).

These results suggest that Cy3 and Cy5 signals on antibody microarrays are stable when the microarray slides are stored in an airtight slide vial at -20°C. These results are crucial to guide contemporary proteomics research involving microarrays. For instance, occasionally microarray slides could not be scanned soon after the experiment due to time restrains. If this is inevitable, microarray slides could be stored and scanned at a later time. Oftentimes, it is determined that signals of some proteins on microarray slides were too strong (e.g. saturated) after data analysis has been done. In such cases, a rescan of microarray slides is usually desirable with a reduced setting of the laser power and/or PMT, so that signal intensities of these proteins become non-saturated and suitable for data analyses.

The signal intensities of microarray spots did not show a significant decrease after repeated scans, suggesting that the employed settings of the laser power and PMT were adequate for multiple scans. We used a confocal microarray scanner in our study, which, due to the confocal nature of the laser beam, kept the bleaching effect of the laser light minimal. However, whether a non-confocal microarray scanner could achieve similar results as a confocal scanner remains to be determined. Also, we tested only Cy3 and Cy5 dyes in the present study. It is possible that other fluorescent dyes may show similar results as those of Cy3 and Cy5.

In theory, the fluorescent signal on microarray slides should either remain the same or decrease in intensity but is not expected to increase over time. However, we had a few slides that displayed higher intensities at a later scanning time point when compared to the intensities acquired immediately after the drying procedure. Two possibilities may be accountable for this phenomenon. Such event could be attributed to the instability of the laser power and/or PMT of the microarray scanner. Another potential source of error may be due to incomplete drying of a microarray slide after the final centrifugation. Any remaining fluid in the slide holder may evaporate and keep the microarray slide humid. The moisture on microarray spots is reduced over time, which leads to a stronger fluorescent signal.

In the present study we employed antibody microarray slides from Clontech Laboratories Inc. (Mountain View, California) for the following reasons: The Clontech™ antibody microarrays detect a wide variety of cytosolic, membrane-bound, and nuclear proteins. Over 500 proteins can be examined in a single experiment. As part of the antibody microarray development, all antibodies have been extensively tested to verify their specificity. Each is raised against a known protein. Antibodies that display a poor specific signal or a high level of cross-reactivity were excluded from the antibody microarrays. In addition, all antibodies were checked for the linearity of signal that can be obtained, a necessary step to ensure accurate quantification. Antibodies that show non-linear binding kinetics were also excluded from the antibody microarrays. Each antibody is double printed side-by-side on the microarray slide to provide an additional internal control. The antibody microarrays are produced on standard 75 × 25 × 1 mm glass slides, an open platform that is compatible with commercially available scanners commonly used for DNA microarrays. Thus, the Clontech™ antibody microarrays represent the most comprehensive antibody microarrays to this date. Since the coupling of cyanine dyes (N-hydroxysuccinimide-esters) to proteins is universal through amines, our results should be applicable to other types of antibody or protein microarrays as well.

A recent study showed that ozone levels in the laboratory atmosphere could adversely affect intensities of fluorescent dyes such as Cy5 and Alexa 647 on DNA microarrays [29]. The fact that fluorescent intensities did not show significant decrease at day 10, 20, and 30 when compared to those at day 0 suggests that the ozone level in our laboratory is not a critical component in our antibody microarray experiments.

Based on our experience we summarize the key issues to maintain the stability of fluorescent signals on microarray slides as follows: 1) to dry the microarray slides thoroughly, 2) to keep them under a dry condition, 3) to store them in a freezer (e.g. -20°C), and 4) to minimize light exposure.

## Conclusion

Microarray spot signals are more stable than previously thought if microarray slides are stored at -20°C in an airtight slide vial. Microarray slides can be preserved and rescanned multiple times using a confocal laser scanner over a period of days or weeks.

## Methods

Proteins extracted from visual cortex of young and adult mice were used in this study. The use of animals and the experimental procedures involving animals were approved by the Animal Care and Use Committee of Wake Forest University Health Sciences (Protocol number A03-045). All animals were euthanized with an overdose of pentobarbital (150 mg/kg body-weight). A conventional two-color dye-swap protocol was used for antibody microarray experiments [11,17,18,20,21]. In brief, proteins were extracted using a protein extraction buffer (Clontech, Mountain View, California), which contained non-denaturing detergents. Tissue samples were homogenized with alumina (0.5 g/100 mg tisue) and extraction buffer (2 ml/100 mg tissue). The suspension was centrifuged at 10,000 × g for 30 minutes at 4°C. The supernatant was collected and its protein concentration was measured using a Protein Assay Kit (Pierce, Rockford, Illinois). The protein concentration was diluted to 1.1 mg/ml by adding an appropriate volume of the extraction buffer. The monoamine reactive dyes Cy3 and Cy5 (Amersham, Piscataway, New Jersey) were dissolved in 110 μl extraction buffers, respectively. 50 μl dye solutions and 450 μl protein solutions were mixed to generate 4 samples: Adult-Cy3, Young-Cy3, Adult-Cy5, and Young-Cy5. After 90 minutes of incubation on ice, the labeling process was stopped by adding 4 μl of 1 M ethanolamine. After protein labeling, unbound dyes were removed by gel filtration using disposable PD-10 desalting columns (Amersham). Each column was equilibrated with 3 × 5 ml desalting buffer (Clontech) before adding a protein sample, which was eluted by applying 2 ml desalting buffer. The protein concentration in each sample was determined using the Pierce Protein Assay Kit.

Two antibody microarray incubation solutions were made with the following compositions: (1) 5 ml incubation buffer (Clontech), 25 μg of the Adult-Cy3 protein sample, and 25 μg of the Young-Cy5 protein sample, and (2) 5 ml incubation buffer, 25 μg of the Adult-Cy5 protein sample, and 25 μg of the Young-Cy3 protein sample. After 30 minutes incubation at room temperature with gentle rocking, the antibody microarray slides (Clontech) were washed with seven different wash buffers (Clontech) at 5 minutes each, dried by centrifugation (1,000 × g) in a swing bucket rotor at room temperature for 25 minutes, and scanned using a confocal microarray scanner (ScanArray Gx) with a pixel resolution of 5 μm. The excitation wavelength and the emission filter wavelength for Cy3 and Cy5 were preset by the scanner's manufacturer (Perkin-Elmer, Shelton, Connecticut) at 543 nm/570 nm (excitation/emission) and 633 nm/670 nm (excitation/emission), respectively. After each scan, the microarray slide was put in a dry and air-tight plastic vial, and stored in a -20°C freezer until the next scan. Fluorescent signals of microarray spots were quantified using ScanArray Express (Perkin-Elmer). The mean intensity of the fluorescent signal within each microarray spot as well as the mean background intensity immediately surrounding the microarray spot was determined. The intensity of a microarray spot is defined as the value of the spot mean intensity minus the background mean intensity. Analysis of variance (ANOVA) was applied to test statistic significance of signal intensities at different time points, using signal intensities of the microarray spots as dependent variables and the microarray spots (1014) and time points (4) as independent variables.

## Competing interests

The author(s) declare that they have no competing interests.

## Authors' contributions

QG planned the experiments, participated in the experiments and data analyses, and wrote the manuscript. TMS participated in the microarray experiments and conducted data analyses. CK participated in data analyses.
